# Daptomycin for methicillin-resistant *Staphylococcus epidermidis *native-valve endocarditis: a case report

**DOI:** 10.1186/1476-0711-9-9

**Published:** 2010-02-18

**Authors:** Marylene Duah

**Affiliations:** 1 Samaritan Medical Center, Watertown, NY, USA

## Abstract

Coagulase-negative staphylococci (CoNS) have been increasing in importance as a cause of native valve endocarditis (NVE). Most cases of NVE caused by CoNS are attributable to *Staphylococcus epidermidis*. NVE caused by CoNS acquired in a nosocomial setting may differ from cases acquired in the community in several ways. It may be associated with hemodialysis, the presence of a long-term indwelling central catheter or pacemaker, or a recent invasive procedure; nosocomial cases may have a higher rate of methicillin resistance among CoNS isolates, and so be more likely to be treated with vancomycin. Unfortunately, NVE caused by methicillin-resistant CoNS has been associated with significantly higher rates of persistent bacteremia and in-hospital mortality than methicillin-susceptible isolates. The poor outcomes in these cases point to the need for alternative therapies with potent activity against methicillin-resistant CoNS. In our medical center, a 76-year-old man presented with native-valve endocarditis and positive blood cultures for methicillin-resistant *Staphylococcus epidermidis *(MRSE). During each of three 6-week courses of treatment with vancomycin, blood cultures were negative, but they once again became positive for MRSE when vancomycin was discontinued. The minimum inhibitory concentration of the MRSE isolates for vancomycin remained stable at 2 μg/mL. Eventually, treatment with daptomycin was initiated (500 mg [7 mg/kg]) 3 times/week for 6 weeks. Over the following year, no positive cultures for MRSE were detected.

## Background

Coagulase-negative staphylococci (CoNS) have been increasing in importance as a cause of native valve endocarditis (NVE) [1-3]. According to recent data from the International Collaboration of Endocarditis-Prospective Cohort Study (ICE-PCS), CoNS account for 7.8% of all cases of NVE and cause death in one quarter of these cases, even though a majority of patients undergo surgical treatment [4]. In the ICE-PCS cohort, which included 1635 patients from 61 centers in 28 countries with definite NVE and no history of injection drug use, the mortality rate for NVE caused by CoNS was comparable to that for NVE caused by *Staphylococcus aureus *and significantly higher than that for NVE caused by viridans group streptococci [4].

Most cases of NVE caused by CoNS are attributable to *Staphylococcus epidermidis*, including 80% of those in the ICE-PCS cohort [4]. In this cohort, NVE caused by CoNS acquired in a nosocomial setting differed from cases acquired in a community-based setting in several ways. Nosocomial cases were associated with several predisposing factors, including hemodialysis, the presence of a long-term indwelling central catheter or pacemaker, or a recent invasive procedure [4]. Notably, nosocomial cases had a much higher rate of methicillin resistance among CoNS isolates (58 versus 22%; P = 0.05), and consequently were more likely to be treated with vancomycin (65 versus 25%; P < 0.01). In turn, NVE caused by methicillin-resistant CoNS was associated with significantly higher rates of persistent bacteremia (25 versus 9%; P = 0.01) and in-hospital mortality (40 versus 16%; P = 0.03) than methicillin-susceptible isolates. The poor outcomes in these cases point to the need for alternative therapies with potent activity against methicillin-resistant CoNS.

Daptomycin is a novel cyclic lipopeptide with activity against most aerobic Gram-positive pathogens, including strains resistant to methicillin, vancomycin, and other antibiotics [5,6]. Daptomycin is highly active against CoNS, including methicillin-resistant *S. epidermidis *(MRSE) [7-9]. Most clinical isolates of MRSE and other methicillin-resistant CoNS are susceptible to daptomycin at a minimum inhibitory concentration (MIC) of 0.5 μg/mL or less [10,11]. Daptomycin produces rapid, concentration-dependent bactericidal activity, but its mechanism differs from other antibiotics; it damages the bacterial cell membrane but causes only minimal cell lysis [12-14].

Daptomycin is approved by the US Food and Drug Administration for the treatment of bloodstream infections, including right-sided infective endocarditis caused by methicillin-resistant and -susceptible strains of *S. aureus *as well as for complicated skin and skin structure infections caused by susceptible Gram-positive pathogens [15]. In a randomized clinical trial, daptomycin was as effective as a control regimen (low-dose gentamicin plus either an antistaphylococcal penicillin or vancomycin) in patients with bacteremia caused by *S. aureus*, including the subset with right-sided endocarditis [16] and those with methicillin-resistant strains [17]. A subsequent systematic review of published case reports and case series identified 19 patients with endocarditis who had been treated with daptomycin, mostly after failure of previous vancomycin therapy [18]. In one of these cases, endocarditis with bacteremia was due to CoNS and was treated successfully with daptomycin after vancomycin failure [19]. In an experimental model of endocarditis caused by MRSE, daptomycin produced > 99.9% kill rates by 8 hours after dosing and reduced bacterial load to a greater degree than vancomycin at 1 to 3 days [20].

The preclinical and clinical profiles of daptomycin suggest that it should be useful in treating NVE caused by CoNS, even after the failure of treatment with vancomycin. This report describes a case of NVE caused by MRSE that was treated successfully with daptomycin after the patient failed multiple courses of vancomycin.

## Case Presentation

A 76-year old man receiving dialysis for end-stage renal disease was referred to our medical center because of blood cultures repeatedly positive for CoNS (MRSE). Over the previous 4 months, two infected hemodialysis catheters had been removed, and for each episode, the patient had been treated with a 2-week course of intravenous (IV) vancomycin, with trough drug levels maintained in the range of 15 to 20 μg/mL. After the second episode, blood cultures became persistently positive for CoNS within 1 week of discontinuing vancomycin. A transesophageal echocardiogram revealed moderate aortic valve sclerosis and mild aortic regurgitation and showed stringy masses attached to the aortic cusps suggestive of a bacterial infection (left-sided endocarditis). Low-grade fever was present. The patient reported having some headaches and malaise but denied any chest pain, shortness of breath, or other symptoms. Laboratory test results were: C-reactive protein, 1.9 mg/dL (normal, < 0.5 mg/dL); erythrocyte sedimentation rate, 13 mm/hr; hemoglobin, 13.1 g/dL; hematocrit, 38.9%; platelet count, 281,000/μL; and white blood cell count, 9,000/μL, with a differential of 43% neutrophils, 29% lymphocytes, 16% monocytes, and 10% eosinophils.

### Medical history

The patient's medical history was significant for end-stage renal disease caused by diabetic nephropathy, and congestive heart failure with severely decreased left-ventricular systolic function. The patient had undergone hemodialysis 3 times per week for 4 years. The medical history also showed coronary artery disease, gout, and hyperlipidemia. In 1993, the patient had undergone coronary artery bypass grafting followed by multiple angioplasties, but he was no longer a candidate for cardiac surgery.

### Initial treatment course

**Figure 1:**
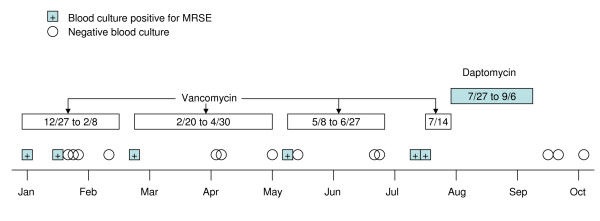
**Timeline showing blood culture results for methicillin-resistant *Staphylococcus epidermidis *in relation to treatment**.

The patient was hospitalized for NVE on December 27, 2006. Two sets of blood cultures were positive for MRSE (Figure 1). Vancomycin 500-1000 mg IV was started after hemodialysis. Repeat blood cultures obtained on January 16, 2007, showed *S. epidermidis *(on one of two sets) that was susceptible (determined by VITEK 2) to trimethoprim/sulfamethoxazole, linezolid, vancomycin, and rifampin. The vancomycin MIC was 2 μg/mL. Rifampin 300 mg bid was added to vancomycin on January 16. Four blood cultures obtained between January 18 and February 8, while the patient was receiving vancomycin and rifampin, were negative. The first course of antibiotics was discontinued on February 8. Twelve days later, on February 20, two sets of blood cultures were positive for *S. epidermidis*. The bacterium was sensitive to linezolid, rifampin, and vancomycin, with the vancomycin MIC remaining at 2 μg/mL.

The patient was started on a second course of IV vancomycin on February 22, while continuing with his dialysis treatment. Trough drug levels were maintained in the range of 15 to 20 μg/mL. The patient had minimal symptoms, except for intermittent low-grade fever and shortness of breath. Blood cultures obtained during the second course of vancomycin were negative. Antibiotics were discontinued on April 30, but 8 days later, blood cultures were again positive for *S. epidermidis*. The patient was started on a third course of IV vancomycin, with a 500-mg dose given after each dialysis session. Trough drug levels were still in the range of 15 to 20 μg/mL. Repeat blood cultures on May 10, June 25, and June 27 were negative. The third course of vancomycin was completed on June 27, but blood cultures on July 12 and July 14 were again positive for CoNS (MRSE). The vancomycin MIC was 2 μg/mL, and the daptomycin MIC was 0.75 μg/mL. On July 14, vancomycin was restarted with rifampin 300 mg three times daily.

### Further treatment course

The patient was admitted on July 27 with fever, chills, and rigor. He was diagnosed with a central-line infection caused by *Enterobacter cloacae *and received lock therapy with IV ciprofloxacin 400 mg daily through his dialysis catheter for 2 weeks. Vancomycin was discontinued. Because the cultures on July 12 and 14 were positive for MRSE, IV daptomycin 500 mg (7 mg/kg) after each dialysis (3 times/week) was started and continued for 6 weeks, until September 6. Blood cultures obtained on September 13 and 20 and October 2 were negative. Over the following year, the patient did not have any recurrence of blood cultures positive for *S. epidermidis*. The patient has remained stable and continues to do well at 18 months after discontinuing daptomycin.

## Discussion

This patient is typical of those who are likely to develop NVE caused by methicillin-resistant CoNS. Consistent with the ICE-PCS cohort [4], the patient had several factors predictive of a poor outcome from NVE caused by CoNS (persistent bacteremia, congestive heart failure, and chronic illness). He also had predisposing factors for methicillin-resistant CoNS (hemodialysis, long-term indwelling catheter) that confer higher mortality. Daptomycin successfully eradicated MRSE after the patient had failed several courses of vancomycin. Although blood cultures were negative during vancomycin treatment, cultures positive for CoNS were seen within 1 week after vancomycin was withdrawn. In contrast, blood cultures were negative during daptomycin treatment and have remained negative for more than 1 year after the 6-week course of daptomycin was completed. The patient was treated with daptomycin at a dose of 500 mg (7 mg/kg) after each dialysis (3 times/week) for 6 weeks, which is generally consistent with the dosing recommendation for daptomycin in patients with bacteremia and endocarditis caused by *S. aureus *[15]. For the approved indication, daptomycin is recommended at a dose of 6 mg/kg for 2 to 6 weeks based on the physician's working diagnosis. Because daptomycin is eliminated primarily by the kidneys, the once-daily dosing schedule should be adjusted to once every 48 hours for patients with significant renal impairment, including those undergoing hemodialysis [15]. In this case, as daptomycin was given with hemodialysis, the drug was given 3 times/week.

## Conclusions

Treatment with daptomycin 500 mg (7 mg/kg) 3 times/week for 6 weeks enabled the patient, a 76-year old man on hemodialysis who had presented with native valve endocarditis, to clear blood cultures positive for MRSE, after 3 courses of vancomycin did not. Treatment with daptomycin offers patients like these an option even after vancomycin failure.

## Consent

Oral informed consent for publication of this case was obtained from the patient. Written informed consent was obtained from the patient's next of kin (his wife) for publication of this case report after the patient expired. A copy of the written consent is available for review from the Editor-in-Chief of this journal.

## Abbreviations

CoNS: Coagulase-negative staphylococci; ICE-PCS NVE: International Collaboration of Endocarditis-Prospective Cohort Study; IV: intravenous; MIC: minimum inhibitory concentration; MRSE: methicillin-resistant *Staphylococcus epidermidis*; NVE: native valve endocarditis.

## Competing interests

The author declares that she has no competing interests.

## Authors' contributions

The author conceived of this case report, gathered the source material, and drafted the manuscript.
